# Old habits die hard: a case study on how new ways of teaching colonoscopy affect the habitus of experienced clinicians

**DOI:** 10.5116/ijme.57d5.5693

**Published:** 2016-09-19

**Authors:** Ole Lund, Berit Andersen, Mette K. Christensen

**Affiliations:** 1 Centre for Health Sciences Education, Aarhus University, Denmark; 2 Department of Public Health Programmes, Regional Hospital of Randers, Denmark

**Keywords:** Continuing medical education, surgery, conscious and unconscious competence, habitus, logic of practice, Denmark

## Abstract

**Objectives:**

The purpose of this study is to explore the habitual constraints and opportunities that affect how experienced clinicians learn new skills and, in particular, how new ways of teaching can influence these.

**Methods:**

We conducted a case study based on a specialized training program for colonoscopy services in Denmark. Data was obtained from a short-term ethnographic fieldwork and in-depth interviews during this program. Participants were 12 experienced colonoscopists and three expert colonoscopy trainers from Denmark and UK. The analysis of data involved categorization, inductive coding, and theoretical reading inspired by sociological theory.

**Results:**

The experienced clinicians' responsiveness to training was shaped by an underlying logic of colonoscopy practice that was characterized by tacit skills, routine work, lower status, skepticism and self-protectiveness. In order to overcome these habitual constraints, the trainers applied a pedagogical approach based on four methods: 1) intellectualization: 'academization' of skills and competencies, 2) sensing and scaffolding: hands-on experiences and learning by doing, 3) asymmetry: accentuating the authority and respect of the trainer, and 4) relation-building: building relationship and engagement between trainer and clinician. This multi-dimensional approach to teaching enabled the trainers to affect the clinicians' logic of practice and to create buy-in (so-called illusio).

**Conclusions:**

Clinical skills include socially constructed behaviors and unconscious competences which affect experienced clinicians' responsiveness to continuing medical education. This study suggests four educational strategies that may help trainers to establish new logics of practice in experienced clinicians and to improve the clinicians' conscious competence.

## Introduction

Considering the rapid change in knowledge, procedures, and technologies in the healthcare sector, it seems pivotal for clinicians and other healthcare professionals to be able to change routines and unlearn old habits. However, the training of experienced clinicians for the purpose of unlearning old habits, learning new skills, and improving conscious competence may pose a considerable pedagogical challenge to trainers, because they have to overcome incorporated routines and established practices. Exploring the habitus of the experienced clinicians may help trainers to understand why some clinicians might instinctively dig in their heels when faced with new teachings. This study provides in-depth insights in the interconnection between the habitus - the socially constructed behaviors and traditions - among experienced colonoscopists and the pedagogical investments among expert trainers in their endeavor to change old habits in the colonoscopists.

Surgery and the techniques of examining and treating the inner structures of the body is a craftsmanship. The craftsmanship of colonoscopy (in short CSPY) is the careful examination of the inner lining of rectum and colon by use of a slim, flexible tube with a small video camera called a colonoscope. This particular craftsmanship is a highly complex skill that takes years to master.^1^^,^^2^ Expert clinicians get to the cecum more often, use less sedation, cause less discomfort, achieve a better patient experience, and find more polyps.^3^^,^^4^ However, when a clinician masters CSPY, he or she often becomes unconsciously competent when performing the procedure, i.e. the clinician may perform it without conscious thought,^5^ because the competency has become a tacit routine and an ingrained habit. But how does unconscious competence influence experienced clinicians' learning?

It has been argued that tacit knowledge and intuitive expertise are the most desirable forms of knowledge in a craftsmanship such as surgery.^6^ Tacit knowledge allows the clinician to act quickly and deal with difficult situations. But when new techniques arise and old habits need change, it challenges and perhaps even threatens the intuitive expertise and unconscious competent craftsmanship of the experienced clinician. Old habits die hard, and it is unclear how the ingrained habits of experienced clinicians can be overcome and improved.

In this article, we explored the habitual constraints and opportunities that affect how experienced clinicians learn new skills and, in particular, how new ways of teaching CSPY can influence these. We believe it important to explore habitual constraints in response to opportunities - in short, the so-called habitus - of the experienced clinician as this will help us understand why some clinicians might instinctively dig in their heels when faced with new teachings and when they train their colleagues. Furthermore, we believe it to be important to investigate the pedagogical approach and teaching methods of experienced trainers who try to change old habits of experienced clinicians, as this will help us to optimize the training of future trainers.

Teaching and learning the skills of CSPY is a growing research field which include a broad range of studies such as measurement of competency in CSPY,^1^^,^^7^^,^^8^ simulation training versus hands-on or bedside training in CSPY,^9^^,^^10^^,^^11^ and trainees' view on teaching in clinical endoscopy.^12^ Most of these studies concentrate on trainees and residents training to be competent and independent clinicians. A few studies concern quality in CSPY.^2^^,^^13^ Based on a thorough inspection of the concept of quality assurance of colorectal cancer screening, Valori and colleagues concluded that "quality is a multi-dimensional concept that requires continuous monitoring of a variety of indicators and a culture of excellence supported by effective clinical and managerial leadership".^3^ The authors stress the importance of the quality of training of novice clinicians as well as that of continued training of independent practitioners and experts. The above-mentioned studies, which are mainly focused on trainees, may not be applicable to experienced colonoscopists.

To the best of our knowledge, only a few studies have explored the continued training of experienced clinicians.^3^^,^^7^^,^^14^ Research in training experienced surgeons indicates that being an experienced clinician in itself may not equip the individual to become a responsive learner of new skills nor a successful educator of novice clinicians.^15^ Moreover, professional values and the traditions of CSPY practice may be influenced by local and national cultures and habits, and these cultures and habits most likely shape the "cultivation" and training of clinicians.^16^ In particular, the training of experienced clinicians for the purpose of unlearning old habits, learning new skills and improving conscious competence may pose a considerable challenge to trainers.^5^ Likewise, the British sociologist Nick Crossley^17^ described the way in which habitual ways of thinking and acting, or what might be called the operations of the habitus, fundamentally influence human activity such as learning and performing skills.

Based on this, our study aimed to explore two interrelated aspects of the training of experienced clinicians: the way in which the experienced clinicians' habitus influence their acquisition of new skills, and the pedagogical investments and teaching approach of experienced trainers when attempting to enable experienced clinicians to establish new techniques.

### Context of the study

Our study was carried out in the context of the Danish health care system. In 2014 a national colorectal cancer screening program was rolled out in Denmark. All citizens aged 50-74 years regularly receive an invitation with equipment to obtain a stool sample. This sample can be obtained at home and mailed to the laboratory for analysis for traces of blood. If analysis of the stool is positive, a CSPY is offered to the citizen with the aim of detecting colorectal cancer at an early stage and to detect and remove adenomas. This requires increased CSPY capacity and in order to ensure high quality CSPYs along with the higher demand of the procedures, Central Denmark Region (one of five administrative units in Denmark with responsibility for healthcare, hospital services, health insurance, general practitioners and specialists) launched a highly specialized training support and development program (the Training the Colonoscopy Trainer program, henceforward: The TCT program) for CSPY services.^3^ This program aimed to improve training provision for a new generation of clinicians and improve the quality of established clinicians in Denmark. The program consisted of three two days courses: 1) CSPY skills improvement training, 2) Basic and advanced polypectomy, and 3) Training the CSPY trainers. As emphasized by the program provider, an essential part of the clinicians' training was to develop their conscious competence, i.e. their explicit understanding of how they perform CSPY, in order to develop a cohort of 12 expert clinicians who will train the next generation of clinicians to the highest international standards.

### Conceptual framework

This study was inspired by sociological theory and embedded in the concepts habitus and illusio.^17^^,^^18^^,^^19^ The concept of habitus can be considered a term for an experienced clinician's dispositions, the underlying and tacit logics of practice that he or she live by, and for the way in which this clinician inhabits the environment in which he or she works. According to Crossley:^17^

"An agent's habitus is an active residue or sediment of his past that functions within his present, shaping his perception, thought, and action and thereby molding social practice in a regular way. It consists in dispositions, schemas, forms of know-how and competence, all of which function below the threshold of consciousness".

In this article, we regard habitus as a dynamic and fluid internalization of skills, cultural norms, values and logics, and as "a structure-in-process"^19^ that is developed by, and develops in the individual's interactions with both others and the environment. Since large parts of the habitus are affected by social and cultural norms, habitus is partly shared with, for example, one's countrymen, family or colleagues. Thus, habitus is not to be understood only as an individual phenomenon, but as a socially constructed practical sense and a practical identity that we share with similarly disposed groups of people. For example, habitus is the practical sense and the practical identity that feels comfortable, right, useful and valuable for a group of clinicians in a professional context.

Learning requires the learners' commitment to, and immersion in the practice that they attempt to master, and it requires that the learners find the practice meaningful and appealing. The sociological concept of illusio describes the appeal that a field of practice, such as CSPY, can have on its participants and the investment that these participants 'capitalize' in the field.^18^ Illusio comes from the Latin term illūdere, which means to create an illusion or make a game of something. A game is only a game as long as the participants maintain the illusion of the game; they must make-believe in a way which involves the systematic failure to recognize that what they are doing is just playing a game,^18^ no matter how serious it may be. Playing a game involves adhering to rules, virtues, sayings, and doings of the particular game. Thus the concept of illusio may explain how a learner's immersion in a field of practice requires that the learner buys into and internalizes the underlying logics of this particular practice and becomes caught up in and by the game.^20^ The learner's interactions with and relationship to other participants within this practice determines the learner's investment and immersion in the practice,^17^^,^^18^ which suggests that motivation to learn is not just a mental condition inside the learner's head, but is also being developed (or at worst reduced) in the interactions with co-learners, trainers etc.

From this perspective a successful learning situation, that is a learning situation that results in the development of desirable knowledge and skills in the learner, must teach the learners the underlying logics of the practice - the illusio - that is to be learned. This will make the learning situation meaningful and appealing to the learners. Or in other words: the learning situation becomes a game worth playing. Thus a successful learning situation and the successful trainer create buy-in among learners and develop an illusio which enables the learners to concentrate all their resources on completing the task of the situation in a particular way. However, learning situations often confront learners with ways of doing things which the learners do not inhabit and therefore presuppose an underlying logic of practice that the learners are not yet familiar with. In these situations the learners might have difficulty adapting new knowledge and skills to their own logics of practice, because they do not buy into the illusio needed for valuing this alternative approach. As an example, a disrupted illusio may result in an erosion of professional capacity.^21^ Thus one important task of trainers is to develop the learner's experience of illusio and thereby facilitate a match between the learner's underlying logic of practice and the logic of practice of the teaching situation. On this basis, it seems pivotal to explore the often unnoticed interconnection in continued medical education: the habitus in the experienced clinicians and the pedagogical investments and teaching methods among experienced trainers.

## Method

In order to ensure transparency and reliability, we provide a detailed description of how this study was carried out.

### Study design and participants

The design of the study was an instrumental case study as described by Robert E. Stake.^22^ Stake argues that case studies, in general, have limited capacity to provide the basis for generalization. On the other hand, case studies of an instrumental kind which have a general research question or issue as their focal point which we seek insight into by studying a particular case, may provide a general understanding of the question or issue in focus. In the design of this case study we were using the particular case of a training program (see below) to establish a general understanding of implications of training the expert to teach. As such, the case study was an instrumental case study because we observed the characteristics of an individual unit (the training program) in order to approach a general understanding of the two research questions in focus.

Participants were 12 experienced clinicians (of which 11 were surgeons, 1 was a gastroenterologist; 9 were male and 3 were female). The participants were selected by the head of the four colorectal departments that take part in the colorectal cancer screening program in the Central Denmark Region. The heads of the units were asked to select the most experienced clinicians in their department. Thus our study unit in the case study was the 12 participants and their 3 trainers in the three courses of the TCT program. We collected data from two sources: field work during the three courses and in-depth interviews with 10 of the 12 participants. Prior to participation in the study all participants were informed about the study verbally and in writing, and they signed a written statement of consent. The participants were anonymized in the presentation of data.

The authors obtained permission from the Danish Data Protection Agency to use and combine the specific data generated in the field work and interviews for the purpose of this study as required by Danish law. In concordance with the guidelines of the Regional Ethics Committee in Denmark,^39^ the study was exempt from ethical approval due to its qualitative design.

### Data collection methods and procedures

Data for the case-study was obtained from a short-term ethnographic field work^23^ and in-depth interviews.^24^ The principal author of this article conducted the fieldwork for ten days of observations during the entire TCT program held on three occasions in April, June and October 2014. He participated as a passive participant^25^ which meant he was present as a bystander at course activities such as introductory dinner, class-room based sessions, demonstrations, supervision and case-training sessions, debriefings, evaluations, lunches, coffee breaks etc. In addition, he took part in several informal conversations with the participants and the trainers. He aimed at gaining insight into the atmosphere in which the training was carried out by attending to the sensoriality of practice.^26^ Fieldnotes were taken at the time, mostly in the form of cues and short sentences, and these were further developed into more detailed descriptions within hours after having made each observation. The detailed field notes (55 double-spaced pages) included descriptions of transpiring events, moods, conversations, ways of interacting, gestures, use of voice and reflections on what educational strategies that were put in use in the different training situations. Also included were the ways in which the training situations developed the participants' investment in and feel for the procedures and methods of the trainers.

In between the 2^nd^ and 3^rd^ training session in TCT program we conducted in-depth interviews with ten clinicians. In advance, we prepared a semi-structured interview guide^24^ based on preliminary data from observations of the first two training sessions. The purpose of the interviews was to invite the participants to elaborate on how they experienced the training during the courses, and how they experienced and were affected by the trainers' way of training them. As was the case with the fieldwork, the interviews were carried out by the principal author, and this provided the dialogues in the interviews with actual and shared situations from the observations as a concrete frame of reference during the interviews. The principal author interviewed the participants in their respective work places, and he applied the same semi-structured interview guide to all ten interviews. The interviews were audiotaped and the recordings lasted a total of six hours.

We organized and coded the collected data from the field work and in-depth interviews in 13 data units: 3 sets of field notes and 10 interview recordings. The 13 data units were organized in NVivo 10. This software allows a researcher to combine field notes with audio recordings of the interviews and code them in the same program.

### Data analysis

We reviewed the data units several times and conducted the analyses in three steps. First we applied nine codes in a deductive categorization of data in order to organize data according to generic educational strategies in medical education.^5^ The categorization revealed the range of educational strategies among the participants and provided an overview of most common strategies. Secondly, we conducted inductive analysis of data in order to expand our interpretation of data. We found six themes that were not captured by the deductive categorization but which stood out as influential to the clinicians processes of learning, for example cultural barriers and the significance of old habits. This analysis emphasized that the training of the experienced clinicians was not just a question of educational strategies introducing the clinicians to new scoping techniques, but a lot of the training involved dealing with and impacting underlying forces and motives that guided the clinicians' practices in order to make them responsive to the technical teachings.

As a consequence of the insights we reached during the first and second steps of the analysis, we proceeded to the third and final step where we conducted a theoretical reading^24^ of the most recurring codes in the data material, i.e. codes with more than 30 quotations distributed over more than 10 data units. The theoretical reading was conducted from the perspective of the sociological concepts of habitus and illusio as already described. We applied these concepts to facilitate interpretations of the way in which the learning situations created buy-in among clinicians. This process resulted in two dominant themes which synthesized the main outcome of the data analysis.

The interpretation of data was developed and validated in several ways: 1) in processes of researcher triangulation during study group meetings between the three researchers, 2) in processes of participant triangulation during informal talks with the participants during the field work, and 3) by presenting the preliminary interpretations of data to the participants after the TCT program and inviting them to comment on this.^27^ Furthermore it was of great importance that the interpretations happened in conjunction with the insider view and embodied, sensorial, and emotional knowing^26^ that the principal author had developed by being close to the participants' training during the courses. In the process of analysis, researchers risk abstracting themselves from the field of research and over-interpreting and simplifying the meaning of data. Thus we aimed at upholding the connection to practice via embodied triangulation, i.e. continuously testing interpretations against the insider view of the principal researcher.

## Results

The reading and analysis of data revealed two synthesizing themes: 1) The tacit skills and logics influence the clinicians' responsiveness to the training, and 2) Creating buy-in: The multi-dimensional approach of the expert trainers. The complete list of codes, the total number of quotations, and the synthesizing themes across the 13 data units are illustrated in Table 1.

**Table 1:** Results of the three steps of the analysis: codes and dominant themes in the 13 data units (three sets of field notes and ten qualitative interviews)

Deductive analyses resulted in nine codes	No. Data Units	No. Quotations	Dominant theme in the theoretical reading of data
Relation-building: Investment in building relationship and engagement between trainer and clinician	11	64	Creating buy-in: The multi-dimensional approach of the expert trainers
Intellectualization: academization of skills and competencies	11	40	
Asymmetry: Accentuating the authority and respect of the trainer	11	37	
Sensing and scaffolding: hands-on experiences and learning by doing	11	35	
Pointing out failures and giving correctional feedback	9	17	
Guiding and educating the clinicians' attention	8	20	
Identification, imitation and role-modeling	4	17	
Training at the edge of one's comfort zone	4	6	
Concentrated and deliberate focus on improvement	4	5	

Inductive analyses resulted in six codes	No. Data Units	No. Quotations	Dominant theme in the theoretical reading of data
Clinicians are under the influence of their own routines and old habits	11	49	Tacit skills and logics are strongly influencing the clinicians' responsiveness to the training
Clinicians are under the influence of cultural logics	11	43	
The power of repetitions	9	14	
Mirroring habits and routines	8	20	
Constraining structural conditions	7	19	
Developing the colonoscopists' investment and creating illusio	7	16	

First, we describe the theme about tacit skills and logics influencing the clinicians' responsiveness to the training. This theme demonstrates the clinicians' point of departure when they enter the Training the Colonoscopy Trainer program and the way in which it sets the scene for the pedagogical encounter between clinicians and trainers.

Then we describe the theme regarding the multi-dimensional approach of the expert trainers and how the trainers create buy-in. In this theme we establish a model for the multi-dimensional approach of the expert trainers. The model illustrates in which ways the trainers manage to create buy-in among the clinicians - despite the clinicians' habitual constraints.

### Tacit skills and logics influence the experienced clinicians

The interviews revealed that experienced Danish clinicians were accustomed to engaging in a specific cultural logic of colonoscopy practice, which was based on underlying and unwritten rules and conventions. This logic encouraged the clinicians in Denmark to carry out CSYP in a certain way that we may call the Danish scoping logic that was characterized by tacit skills, routine work, lower status, and self-protectiveness. The following analysis and selected quotations will substantiate and develop this result.

### Tacit skills

Our data demonstrated that the clinicians relied heavily on tacit skills while scoping. This means that, over time, they have let the details of the technique utilized for manoeuvring the scope fade into the background of their attention. This was desirable because it allowed the clinicians to concentrate on e.g. finding their way in the bowel and searching for polyps. But at the same time, this might restrict them from being aware of and articulating what and why they do what they do.

"During the training program [we were asked to reflect on] what it is exactly that makes us advance in the bowel. And this is something that I'm not used to think about in my day-to-day work. We have often more or less randomly turned the scope a little bit and - in our department - we have given the patient more sedation." (Surgeon 8, male chief consultant)

"So we have just done something which our experience tells us is right. But we haven’t thought about why this is so." (Surgeon 6, male chief consultant)

Furthermore, one of the clinicians indicated that the incentives to reflect on habits and logics of practice decrease when you have reached a certain level of experience and responsibility at the ward:

"We have reached a point in our career in which we are not used to being supervised. [...] When we operate, scope etc., we are often the one in charge." (Surgeon 9, male chief consultant)

Overall, the study showed that a culture has developed in the Danish context of CSPY that discourages the clinicians from questioning and inspecting their ingrained habit and logic of practice. The discouragement was developed early in the clinicians' career in the sense that CSPY was usually taught 'silently'. More experienced clinicians showed their younger and less experienced colleagues how they perform CSPY without accompanying explanation. The clinicians in this study emphasized that they 'repeat' this way of teaching CSPY because "I can't explain what I do" (Surgeon 2, male specialist doctor).

### Routine work such as CSYP has lower status

Our study also detected a second logic of practice that guided the clinician’s work. They were used to perceiving CSPY as routine work and they did not ascribe it the same status as open surgery.

“We are surgeons […] not endoscopic surgeons! To a surgeon, surgery prevails. This is how it ought to be. That’s how I think it should be. So you might have a tendency to downgrade CSPY […] We have always laughed at the non-surgical clinicians, because they could do four colonoscopies, while we did eight. But you can look at it differently and think: this is wrong if we have done a poor job and overlooked a lot of stuff, while they have done a good work and been careful. But this might be what you can call a ‘Tarzan-attitude’, which you might find among surgeons. We think: how long is this supposed to take? I don’t want to fumble and dawdle at it. Let’s move on!’ We (surgeons) are quite impatient.” (Surgeon 9, male chief consultant)

Among Danish clinicians there seemed to be an implicit agreement to ascribe CSPY low status or even perceived it as “hack work” (Surgeon 9, male chief consultant). This logic of practice discouraged the clinicians from putting their full investment to work. Furthermore, this underlying logic encouraged the clinicians to speed up their scoping:

“10:51 AM. During a case training session one of the clinicians (Surgeon 4, male chief consultant) clearly is used to scope in a fast tempo. It is difficult for him to advance the scope as slowly as the trainer would like him to. Again and again the trainer stresses the importance of advancing slowly: ‘You have to push the scope as slowly as you have ever pushed the scope!’" (Fieldnotes)

“We are trained in the spirit that it’s all about advancing fast. You are skilled if it takes you two minutes to get to cecum.” (Surgeon 6, male chief consultant)

“We are all aware that every examination and surgical intervention is very important to the patient and time shouldn’t play any big part in this. But when every day you are told that you need to hurry, that there are long waiting lists and increasing number of patients, and that we haven’t got the time to…Well, you hear it so many times that eventually you believe it and align yourself with it. [The interviewer: “And it affects each examination or each operation?”] “Yes it certainly does! You try to save as much time as you can.” (Surgeon 3, female specialist resident)

The above quotations portray an atmosphere of bustle and haste in the wards which was nourished by e.g. long waiting lists, overbooked daily programs, and cut-backs among staff. This atmosphere seemed to have fundamental impact on the individual clinician’s scoping habits.

### Experienced clinicians’ skepticism and self-protectiveness

Furthermore, our study showed that the habitus of the clinicians was characterized by professional pride and deep confidence in one's own abilities. This was reflected in the skepticism and self-protectiveness that the clinicians tended to show in regards to the new ideas and ways of the trainers.

“We are taught to be skeptical when we are introduced to new methods. There are thousands of opinions as to how diseases should be treated and cured, and it is very difficult to say with certainty what the right method is. So we have a healthy skepticism as a starting point. And it is probably reinforced in this program because they introduce a new method in an area where I might not consider myself an expert, but at least as very experienced. And when you are told: ‘What you have done is not the best way to do it. We have a better one!’ Your skepticism will be reinforced - as a starting point!” (Surgeon 8, male chief consultant)

This skepticism and self-protectiveness seemed to arise among the clinicians when they feel their underlying logics of practice are questioned or threatened.  As depicted in the quotation beneath, skepticism might also arise in situations in which one received instruction:

“It might be demanding to receive instruction in Denmark, because receiving instruction is sometimes regarded as criticism. There may be two reasons for that. It may be that the teacher expresses criticism instead of guidance. Or that the learner has so much pride that he or she cannot receive instruction. It's probably a combination of both [...] Sometimes trainees might ask about how they are doing in the training, because ‘nobody says anything.’ When nothing is said, it is because everything is going well!’ [Laughs] [The interviewer: [Laughs] Is silence the most positive feedback one can get?] “Yes, and it is actually quite positive. But perhaps this attitude should change.” (Surgeon 4, male chief consultant)

This suggests that previous training experiences have developed a certain kind of logic of practice among the clinicians which encouraged them to be mainly skeptical when faced with alternative ways of scoping. Furthermore, the Danish tradition of training in the form of criticism has developed an underlying expectation in the clinicians which made them receive feedback as criticism (even in cases where the feedback is not intended as such). At the outset of the training program, these above mentioned characteristics of the clinicians’ habitus seemed to constrain them from being fully responsive to the training of the trainers. In the next section we will show how constraints were met and counteracted by the approach of the trainers.

### Creating buy-in-the multi-dimensional approach of the expert trainers

**Figure 1:**
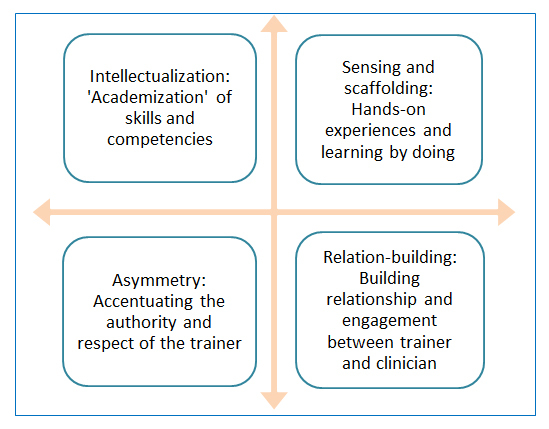
The multi-dimensional approach of the expert trainers in order to create buy-in among the clinicians

Despite the abovementioned constraints the study showed that the pedagogical investments and teaching methods of expert trainers were able to initiate learning, when they in addition to pure technical training also focused on affecting the clinicians’ logic of practice and illusio within the training context. Thereby they focused on developing a deeply felt sense of ‘buy-in’ in the clinicians to the way of scoping promoted and exemplified by the trainers. As illustrated in Figure 1, the multi-dimensional approach of the expert trainers for creating buy-in among the clinicians, this theme represents the interrelationship between four of the most frequently occurring codes in the data material, and thus the four most current and observable pedagogical investments and teaching methods among the trainers.

### Relation-building

The clinicians voiced that the trainers' appreciating way of teaching, i.e. the way the trainers created buy-in among the clinicians by investment in building relationship and engagement between trainer and clinician, was helpful to the clinicians’ own investment in the training activities during the course.

“You sense that [the trainers] are aware that they are talking to someone who is experienced and someone who has been scoping for 25-30 years.” (Surgeon 2, male specialist doctor)

“Of course there are methods that they want us to acquire, but we are allowed to partake with our own knowledge. So it is not kind of imposed on us.” (Surgeon 7, male chief consultant)

“You feel like an equal, even if you are not an equal in the training situations.” (Surgeon 4, male chief consultant)

The trainers appeared attentive and appreciative towards the clinicians' ways of understanding and performing CSPY. As a consequence, the clinicians appeared to be more open to the trainers' ways of doing things than first expected:

“You might become defensive if you are not recognized. If you just are being treated like a little cub. If they meet us with this approach, I think most of us will dig in our heels. But they absolutely did not.” (Surgeon 6, male chief consultant)

This indicates that the trainers' relational approach helped disarm the habitual tendencies of approaching training situations with skepticism among the clinicians. In addition humor played an important part in the trainers’ and clinicians’ way of building relations. As an example:

“During a discussion in class, one of the trainers asks a clinician (Surgeon 7, male chief consultant) a question and the clinician answers what the trainer is expecting. The trainer stretches his arms out toward the clinician as if he is getting ready to give the clinician a hug and exclaims: ‘I could kiss you!’ And everybody burst into laughter when the other trainer continues: ‘Maybe we need to leave these two guys alone!’” (Fieldnotes)

The frequent use of humor and the unpretentious atmosphere seemed to help the clinicians to break away from dogmatic notions about themselves which they were encouraged to live up to in their daily life at the wards. Thus in this atmosphere, a logic of practice was developed which increased the clinicians’ responsiveness to the training by making it easier for them to break away from their professional pride and ingrained skepticism towards new learnings.

“[The trainers] put our feeling into words ... It's like when an author is able to articulate one's feelings in a novel for example. Then you think: ‘Ah, that's a nice description!’ They are able to articulate actions that you haven't been reflexive about. They thought through and uncovered what we have done unconsciously.” (Surgeon 1, male chief consultant)

“He [the trainer] is standing right next to me. And I can feel that he has the experience. And he describes to me what I can feel with the scope, as I'm scoping.” (Surgeon 2, male specialist doctor)

The data showed that the trainers were sensible to the way the clinicians inhabit their practice and able to align themselves with the logics of practice which guided the clinicians’ way of scoping. Moreover, the trainers introduced a shared vocabulary about the individual feeling of scoping. These two capabilities in the trainers convinced the clinicians of the value of being able to perceive and articulate detailed tactile sensations during scoping as a way of building a relation that facilitated a match between the learner’s underlying logic of practice and the logic of practice of the trainers.

### Intellectualization

The trainers were often able to put into words what the clinicians already knew pre-reflectively and managed to hit a nerve in the clinicians that made them grasp the gains of learning to scope the way the trainers suggested. They did this by using an advanced vocabulary about feelings and tacit knowledge, and by using intellectualization and academization of skills and competences related to the clinicians own experiences:

“Their way of explaining their scoping technique, i.e. their ways of turning the patients in order to exploit the distribution of air and fluid in the optimum way, is immediately obvious. And it makes the teachings much more inviting. An experienced surgeon who has made a thousand colonoscopies will naturally have some built-in skepticism when someone thinks that they have found the philosopher's stone. But the way [the trainers] presented and explained their ways made it hard for us to find contradicting arguments that emphasized that this is not worth trying.” (Surgeon 7, male chief consultant)

The trainers' arguments became convincing because they were in keeping with the underlying logics of the scientific realm of understanding that both parties adhered to, for instance when guiding the clinicians' attention to the way gravity influence fluids when arguing in favor of turning the patients more frequently.

### Asymmetry

The use of the underlying logics of the scientific realm of understanding not only gave the trainers' words weight, but it also positioned the trainers as authoritarian role-models in the eyes of the clinicians, and it accentuated the asymmetry between experts and learners:

“They show us the rationale. [...] And every time you ask a question they demonstrate that they have thought things through [...] and they gain authority when we realize that there is no rationale behind our way of scoping. Their ways of scoping seem more academic rather than worker-like.” (Surgeon 1, male chief consultant)

By using a causal and logical line of arguments the trainers also lived up to academic conventions of being reflective. Thus they appeared as authorities in relation to the clinicians, who in comparison felt more like blue-collar workers, who primarily deal with a less reflective practice rather than engage in a critical reflective profession.

But a well-picked word or argument is only part of the multi-dimensional approach of the expert trainers. By virtue of well-orchestrated appearance, and carefully chosen manners and skills, the trainers exposed an extra layer of information about their underlying logics of practice that were at least as efficient as their words in the process of convincing the clinicians to adapt the trainers’ ways of scoping:

“I like their style. And you can tell that they are passionate about it. They are able to express that this is the most enjoyable thing to do, and that you have to put all your efforts into it.” (Gastroenterologist 1, male chief consultant)

Not only did the trainers make the impression of being engaged expert trainers captivating the clinicians with their passion; they positioned themselves as expert clinicians:

“11:19 am. [The clinician (Surgeon 4, male chief consultant) has for over 10 minutes tried to get past an obstacle in the patient's bowel] The clinician is still not advancing. The trainer asks if he shall take over. He takes over and we can see on the screens [in the classroom] that by maneuvering the scope slowly, he advances quite quickly [...]The trainer hands the scope back to the clinician while emphasizing that the most important thing is to go slowly.” (Field note)

In situations of shared practical involvement the trainers' habitual behavior, their underlying logics of practice, and their skill levels were concretized to the clinicians. And usually the trainers' skills appeared more advanced and fine-grained. On these grounds the relation of authority between trainers and clinicians were developed further and the trainers’ way of scoping and their underlying logics of practice were emphasized and made desirable among the clinicians.

### Sensing and scaffolding

As shown in the above section, a general and repeated instruction from the trainers to the experienced clinicians was to “slow down” when advancing the scope. The words “slow down” almost became a shared refrain during the program, because a slower pace during scoping increased the clinicians’ sensitivity to the functional relationships between scope-movements and the patients’ intestine, and enabled the trainers to scaffold the clinicians’ practical sense. 

All the clinicians expressed that the hands-on experiences guided by the trainers were crucial for developing the abovementioned desire towards the trainers’ more sensitive ways of scoping:

“To me it is still crucial to have hands on [the trainers' ways]. The feedback I get from the scope when I make certain movements or when feeling the adjustment options enables me to acquire the principles behind [the trainers' way].” (Surgeon 7, male chief consultant)

“You get the effect right away, right? You realize that ‘hey, when I twist the scope the way he wants me to it helps and I advance. And if I turn the patient around, water and air is distributed the way [that they have told us].’ So this is convincing and immediate feedback that goes right in. You cannot get more direct payoff. You realize when you do this [turn the patient around]: ‘Hey yes, damn it helps!’” (Surgeon 6, male chief consultant)

During the slow-paced case-training the verbal descriptions and guidance by the trainers affected the sensations and actions of the clinicians. Furthermore, our observations clearly revealed that the ideas and suggestions of the trainers were consolidated when the clinicians actively engaged in these while solving concrete practical problems, for example when the clinicians came to realize that turning the patients (a method that was highly unfamiliar to the clinicians because it was assumed to delay the procedure) eased the advancement of the scope, and gave a better overview of the bowel. Due to the combination of guidance and direct sensuous and bodily ‘feedback’ afforded by the slower pace and the trainer’s scaffolding, the clinicians received deep layers of information about the trainers' way of scoping which they probably never would obtain from entirely disconnected descriptions (no matter how rich these descriptions would be). In this way, the slow-paced case-training rendered it possible to overcome the ‘Danish scoping logic’ and initiate the refinement of the clinicians’ scoping habits.

## Discussion

The aim of this study was to explore two interrelated aspects of the training of experienced clinicians: the way in which the experienced clinicians’ habitus influence their acquisition of new skills, and the pedagogical investments and teaching methods among experienced trainers when attempting to enable experienced clinicians to establish new techniques. We found that Danish clinicians’ habitus was characterized by tacit skills, the perception of CSPY as routine work with lower status than surgery, and a strong skepticism towards corrective advice and feedback. We also found that expert trainers were able to deal with these constraints when being occupied with building relations, using intellectualization and academization of skills and competences, positioning themselves as authoritarian role-models, and scaffolding the clinicians’ practical sense of scoping.

### Clinical skills are socially constructed

One of the main findings of our study is that clinical skills are socially constructed and highly influenced by the underlying logics of practice in a given context. Thus our finding challenges previous studies which seem to regard surgical competencies and clinical skills as individual properties that can be described independently of organizational structures and social contexts and interactions.^28^^,^^29^^,^^30^^,^^31^ However, according to de Cossart and Fish,^16^ the actual ‘doing’ in surgery is only the 10% tip of the iceberg of professional practice: 

“The visible performance is underpinned by a range of invisible but highly significant resources and underlying drivers, the foundation of which is values”.

Accordingly, we suggest that the clinicians’ scoping habits are not individual patterns of habit but must be understood as a shared habitus closely tied to the environment and the culture in which the clinicians practice on a daily basis. The practice of colonoscopy becomes relevant and meaningful to the clinicians on the backdrop of shared - underlying and tacit - ‘logics’ which guide their ways of committing and investing themselves in their practice.^17^^,^^18^ Due to the reproduced and confirmed logics of practice in their daily work environment, the clinicians are almost automatically ‘pulled towards’ a certain way of scoping and thus caught up in a particular illusio. In the case of the Danish clinicians in this study, the shared logics of practice seem to constrain the clinicians’ opportunities to learn new skills and approaches to the procedure. The clinicians’ habitus work as a barrier to reach expertise, because their locally and socially accepted level of performance seems to be arrested in its effortless automated form.^32^ Thus our study affirms psychologist KA. Ericsson’s case^33^ that;

“the key challenge for aspiring expert performers is to avoid the arrested development associated with automaticity and to acquire cognitive skills to support continued learning and improvement”.

In the following section we discuss the manners in which the professional trainer may release the clinicians’ arrested level of performance.

### The professional trainer: medical expert and master craftsman

Studies on colonoscopy training often focus on listing the technical and cognitive skills that are required by younger trainees in order to perform colonoscopy safely, effectively and comfortably.^8^^,^^9^^,^^11^^,^^34^ These studies pay less attention to the way the trainees’ habitus influence the training process and therefore risk advancing the presupposition that the trainees (and certainly experienced trainees) can be regarded as 'clean slates' that may be taught new technical skills in a straightforward manner. Contrary to these studies, our findings show that the scoping habits of the trainees are influenced by a tacit rationale developed through past practical experiences which affect the trainee’s responsiveness to training. This suggests that trainers and faculty developers may need to consider the professional identity and habitual background of the clinicians, and that the pedagogical investments of the trainers should include not only the development of technical skills, but also relation-building and establishing authority both as a medical expert and a professional trainer.^5^ In short, our study underpins the need for a learner-centered approach to teaching^35^ especially when the learner is an experienced clinician. According to constructivist learning theory,^36^^,^^37^ a learner-centered approach includes that 1) the learners feel that their habitual relation to practice is legitimized and taken into consideration by the trainers, 2) the learners experience that the teaching situation builds on their current habits and advances their performances, and 3) the trainers create buy-in and provide the learners with the access to the trainers’ underlying logics of practice. Our findings clearly support the premise of a learner-centered approach to teaching. More importantly, they also support the importance of the implicit and bodily aspect of creating buy-in which has been less examined in previous studies on teaching and learning in clinical environments.^5^^,^^14^^,^^38^ Instead, these studies suggest that efficient training is characterized by learners investing themselves in the training by focusing on explicit performance goals, thus arguing in favor of a training practice in which learners primarily have an instrumental and rational approach to training. For example, Ericsson’s concept of deliberate practice,^38^ in particular the formulation of clear and appropriate goals for mastering articulated aspects of practice, is acknowledged as a necessary part of performance enhancement.^5^^,^^14^ Goal-setting is undoubtedly important for learning and the motivation to learn. However, our study emphasizes the importance of accompanying goal-setting with scaffolding the experienced clinicians’ practical sense. By scaffolding the clinicians’ sense of practice, the trainers gradually shape the bodily felt sense of the commitment and motivation in the clinicians, and by doing so the trainers attract the clinicians towards the desired scoping habits of the trainers, i.e. the clinicians get a sense of the trainers’ illusio. This insight in teaching and learning clinical skills is rare in the psychologically inspired medical education literature,^5^^,^^16^ and so a sociological conceptual framework including habitus and illusio^17^^,^^18^^,^^19^ such as the conceptual framework of this study, seems appropriate in order to explore the social and bodily understructure of a learner-centered approach to training of experienced clinicians.

### Limitations

The design of this study was a short-term ethnographic field work and in-depth interviews during a period of six months, and so the design did not include a longitudinal follow-up investigation. Thus we are not able to explore the long-term effects of the training program and the clinicians’ ability to employ their renewed habits in the daily workplace environments.

A recent study on faculty development in surgical specialties suggested that “long-term interventions provide more sustained change in learning, transfer of skills to the educational environment and long-term organizational change”.^15^ Our study substantiates why continual and longitudinal training is needed since the results emphasize that the clinicians’ responsiveness to training is dependent on their already incorporated habitus. According to the theoretical framework of this study, habitus is to be understood as a socially constructed practical sense and a practical identity that is shared with similarly disposed groups of people. This means that changing a clinician’s habitus includes organizational change. So an important question remains to be answered: Are the skills and buy-ins developed during the training program maintained and transformed into a sustained habitual change when the clinicians’ renewed habits are no longer supported by the context of the program and the close interactions with the trainers? Unfortunately, this question cannot be answered within the scope of this study.

Finally, the distribution of male and female participants in the study was not representative of the Danish population of clinicians within colonoscopy, but was a result of an internal selection process in Central Denmark Region based on criteria such as years of experience and position in the department, and not representativeness. The gender aspect was not included in the analysis of our data, and this means that interesting insights in the study questions may have been omitted.  

## Conclusions

To the best of our knowledge, the majority of research within the area of training and learning new clinical skills focus on students, junior doctors, or residents. Only few previous studies have explored the continuing medical education of experienced colonoscopists who have several years of practical experience.^4^^,^^7^^,^^14^ Our study shows that clinical skills include socially constructed habitus including behaviors and traditions such as notions of status, skepticism, and self-protectiveness which affect experienced clinicians’ responsiveness to training. Old habits die hard, but this study demonstrates that experienced trainers who employ a multi-dimensional approach to teaching are able to overcome the experienced clinicians’ tacit skills and underlying logics of practice. In conclusion, we will advocate four integrated educational strategies that may help trainers to establish new logics of practice in experienced clinicians:

1.     The trainer has a learner-centered approach to teaching.

2.     The trainer stands out as an authority, an academic role-model, and a practical expert in face-to-face interactions with the clinicians.

3.     The trainer considers, recognizes, and actively engages with the incorporated routines and established practices of the clinicians when guiding and arguing for why the clinicians should change their habits.

4.     The trainer encourages the clinicians to slow down and guides them towards sensuous aspects of the trainers’ standards of good work and their underlying motivations.

These modest suggestions may help trainers and faculty developers in establishing learning environments and training contexts with a logic of practice that promote learning by emphasizing the importance of creating buy-in that operates at the most pre-reflective (or we might say: intuitive) level of the experienced clinicians’ aspirations and in this way overcome old habits.

### Acknowledgements

We wish to thank the clinicians and the trainers who kindly gave their time to participate in this study and shared their experiences with us. In particular, we wish to thank Dr. Roland Valori and Dr. John Anderson (Gloucestershire Royal Hospital, UK, and the English NHS) as well as Dr. Knud Thygesen and Prof. Søren Laurberg Aarhus University Hospital, DK, for providing the unique and exclusive access to the training program and its participants, and for an educational mine of insights. Finally, we wish to thank Dr. Thomas Møller Jensen, The regional Hospital in Horsens, DK, for his strong commitment in hosting the training program.

The study was funded by Central Region Denmark and Centre for Health Sciences Education, Aarhus University.

### Conflict of Interest

The authors declare that they have no conflict of interest.
